# Epigenetic modifications and their roles in pediatric brain tumor formation: emerging insights from chromatin dysregulation

**DOI:** 10.3389/fonc.2025.1569548

**Published:** 2025-06-17

**Authors:** Kento Kawata, Owen S. Chapman, Satoshi Narumi, Daisuke Kawauchi

**Affiliations:** ^1^ Department of Pediatrics, Keio University School of Medicine, Tokyo, Japan; ^2^ Department of Neuro-oncology, Institute of Brain Science, Graduate School of Medical Sciences, Nagoya City University, Aichi, Nagoya, Japan

**Keywords:** pediatric brain tumor, epigenetics, chromatin remodeler, histone modification, genomic rearrangement, extrachromosomal DNA (ecDNA), fusion oncogene, DNA methylation

## Abstract

Pediatric brain tumors, the most devastating cancers affecting children, are believed to originate from neural stem/progenitor cells in developing brain. In precise timing and specific regions during the brain development, chromatin deregulation plays crucial roles in redirecting normal neuronal differentiation pathways toward tumorigenesis. Indeed, epigenomic abnormalities are thought to be more important for brain tumor formation especially in children than adults, as pediatric brain tumors generally exhibit fewer genetic mutations compared to adult brain tumors. Given the small number of mutations, targeting such limited alterations involved in cancer epigenomes is expected to be more effective in pediatric brain tumors. The mechanisms of cancer epigenomes include mutation or dysregulation of chromatin remodelers, histone modifiers, histones themselves, and DNA methylation enzymes. Furthermore, genomic rearrangements and/or higher-order chromatin topology also contribute to these epigenomic mechanisms. These mechanisms are commonly observed in various types of pediatric brain tumors. However, alterations in chromatin regulatory factors differ across tumor types, reflecting the unique epigenetic landscapes shaped by their tumor origins. Accordingly, clarifying their functional similarities and differences across tumor types could offer valuable insights for finding new therapeutic strategies. Thus, this review article focuses on elucidating how pediatric brain tumors arise from epigenomic deregulation and what epigenetic molecules or mechanisms could serve as therapeutic targets.

## 1 Introduction

Pediatric cancers develop when cells gain abnormal proliferative capacities due to the disruption of genetic programs for cellular differentiation during development (1, 2). While both familial and sporadic genetic mutations are recognized as primary causes, cancers are not solely driven by mutations in protein-coding genes directly relevant to cell proliferation. Recent cancer genome sequencing efforts have uncovered recurrent mutations in genes responsible for chromatin regulation (3, 4). These findings suggest that cancer progression may require not only aberrant upregulation of the genetic programs responsible for cellular growth signaling but also specific genomic alterations known as the cancer epigenome, which plays a pivotal role in tumorigenesis (5). In fact, pediatric solid tumors generally have fewer genetic mutations compared to adult tumors; therefore, epigenomic abnormalities are believed to be more important in tumor formation in children than adults (1). The same holds true for the differences between pediatric and adult brain tumors (6).

**Figure 1:**
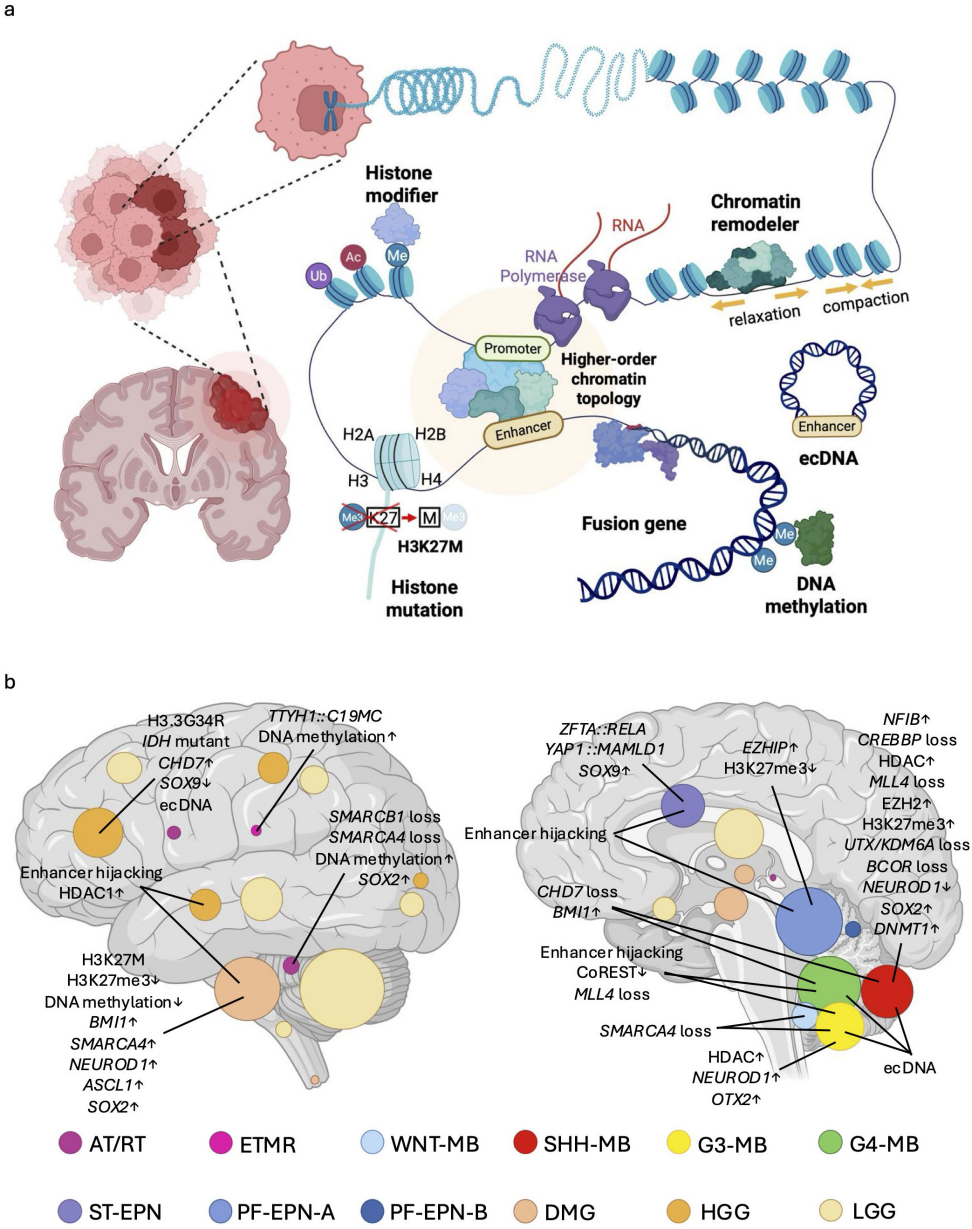
Overview of epigenetic regulatory mechanisms in pediatric brain tumors and their tumor-type-specific distribution. **(a)** Pediatric brain tumors develop through various kinds of epigenetic mechanisms, including dysregulation of chromatin remodelers, histone modifiers, histone mutations, and DNA methylation. Genomic rearrangements may generate gene fusions, extrachromosomal DNA (ecDNA) or alterations in higher-order chromatin topology which also often contribute to the cancer epigenome. **(b)** Schematic illustration graphically depicts the anatomical origins and distribution of each tumor type (26), including Atypical teratoid/rhabdoid tumor (AT/RT) (19), Embryonal tumors with multilayered rosette (ETMR) (25), Wingless medulloblastoma (WNT-MB) (20), Sonic hedgehog medulloblastoma (SHH-MB) (20), Group 3 medulloblastoma (G3-MB) (20), Group 4 medulloblastoma (G4-MB) (20), Supratentorial ependymoma (ST-EPN) (23), Posterior fossa A ependymoma (PF-EPN-A) (23), Circle area is proportional to the number of new diagnoses at each anatomical location. Posterior fossa B ependymoma (PF-EPN-B) (23), Diffuse midline glioma (DMG) (21, 22), High grade glioma (HGG) (21, 22), Low grade glioma (LGG) (24). This image also shows major epigenomic alterations associated with pediatric brain tumors discussed in this study.

The epigenome consists of reversible molecular modifications to genomic chromatin. Chromatin, comprising the ~3 billion base pairs of human genomes bound to histones and other proteins, forms a highly organized structure. Within chromatin, the human genome is efficiently organized and compacted as 146 base pairs of DNA strands wrapped around histone protein octamers, creating repeating units called nucleosomes (7). Such chromatin structures are modified by epigenetic mechanisms including DNA methylation, histone occupancy and modifications, and higher-order chromatin topology (**Figure 1a**). The epigenome plays an essential role in regulating normal cell division and differentiation (8, 9). Disruption of proper epigenomic functions can lead to cellular senescence and/or apoptosis, resulting in developmental abnormalities such as Coffin-Siris syndrome, Nicolaides-Baraitser syndrome, and CHARGE syndrome, which arise from germline mutations in essential chromatin regulatory factors (10, 11). These mutations cause impaired neuronal differentiation, increased cell death, and disrupted neural circuitry, underscoring the critical role of cell-specific epigenomes in maintaining neuronal viability and defining cellular characteristics. Conversely, accumulating evidence indicates that epigenomic changes may also promote abnormal survival and proliferation of brain tumors. Therefore, strategies focused on elucidating mechanisms underlying the cancer-specific epigenome, and subsequent targeting of these alterations, offer promising potential for inhibiting cancer cell proliferation and inducing cancer-specific senescence and apoptosis. Although the path to fully realizing this approach remains challenging, it represents a critical area of research with the potential to lead to innovative therapeutic interventions.

Pediatric brain tumors are the most lethal form of pediatric cancer, arising from both genetic and epigenetic defects during critical stages of brain development (12). Similar to other cancers, global cancer genome sequencing initiatives have identified cancer-specific loss-of-function (LOF) mutations in chromatin-modifying genes, as well as altered genomic rearrangements leading to aberrant epigenetic regulation of cancer-related genes (13, 14). Given that different brain cells at distinct developmental stages give rise to distinct tumor types (15, 16), it is hypothesized that life stage-dependent and region-specific epigenomes underpin the gene expression programs necessary for tumor initiation (17, 18). Thus, it is unsurprising that genetic alterations in chromatin regulatory factors vary across cancer types, reflecting the unique epigenetic landscapes from which these tumors originate (19–26) (**Figure 1b**, **Table 1**). Collectively, it is likely that pediatric brain tumors acquire unique epigenetic regulation that drives tumorigenesis.

**Table 1:** Tumor-type-specific roles of epigenetic deregulation reviewed in this study.

Chromatin remodelers	Tumor types	References
*SMARCB1* loss	AT/RT	(27–29)
*SMARCA4* loss	AT/RT, WNT-MB, G3-MB	(30–32)
*SMARCA4* activation	SHH-MB, DMG	(33–36)
*CHD7* loss	SHH-MB, G4-MB	(37–39)
*CHD7* activation	HGG	(40)
*ASCL1* activation	DMG	(41)
*NEUROD1* activation	G3-MB, DMG	(41, 42)
*NEUROD1* downregulation	SHH-MB	(43)
*OTX2* activation	G3-MB	(42)
*SOX2* activation	AT/RT, SHH-MB, DMG	(44–47)
*SOX9* activation	ST-EPN-ZFTA	(48)
*SOX9* downregulation	HGG	(48)
*NFIB* activation	SHH-MB	(49)
CoREST downregulation	G3-MB, G4-MB	(50)
Histone modifiers		
*CREBBP* loss	SHH-MB	(51)
*HDAC* activation	SHH-MB, G3-MB, DMG, IDH-mutant glioma	(52–60)
*KMT2D(MLL4)* loss	SHH-MB, G3-MB, G4-MB	(38, 61, 62)
*EZHIP* activation	PF-EPN-A, DMG	(63–66)
*EZH2* activation	SHH-MB	(43)
*UTX/KDM6A* loss	SHH-MB	(67)
*BCOR* loss	SHH-MB	(68)
*BMI1* activation	SHH-MB, G4-MB, DMG	(37, 69–71)
Histone mutations		
H3K27M mutation	DMG	(17, 41, 72–78)
H3.3G34R mutation	HGG	(17, 74, 79–81)
DNA methylation		
DNA hypermethylation	ATRT, ETMR, SHH-MB	(82–86)
DNA hypomethylation	DMG	(72)
Genomic rearrangement		
Enhancer hijacking	G3-MB, G4-MB, ST-EPN-ZFTA, PF-EPN-A, HGG, DMG	(38, 87–91)
*ZFTA::RELA*	ST-EPN-ZFTA	(92–94)
*YAP1::MAMLD1*	ST-EPN-YAP1	(95)
*CIC::NUTM1*	CNS ETF-CIC	(96)
*CIC::LEUTX*	Anaplastic pleomorphic astrocytomas, CNS embryonal tumors	(97)
*TTYH1*::*C19MC*	ETMR	(84)
ecDNA	SHH-MB, G3-MB, G4-MB, HGG, spinal EPN	(91, 98–102)

One of the fundamental biological questions that emerges here is whether dysregulation of distinct chromatin regulatory factors across various types of pediatric brain tumors exert similar effects on the epigenome, and what the shared mechanisms and key differences might be. Investigating these aspects could yield valuable insights into the molecular pathways driving tumor formation. However, our current understanding remains limited, and efforts must begin by elucidating the specific epigenetic modifications involved in the formation of individual pediatric brain tumors and their subsequent consequences. Accordingly, this review highlights the molecular mechanisms that influence the epigenome during pediatric brain tumor development.

## 2 Chromatin modifications

Chromatin modifications are essential epigenetic mechanisms that alter chromatin architecture and regulate gene expression. Dysregulation of these processes plays a pivotal role in the pathogenesis of pediatric brain tumors. For instance, chromatin remodelers modify chromatin structure to either open it, forming transcriptionally active euchromatin, or close it, forming transcriptionally inactive heterochromatin, by depositing, removing, or shifting nucleosomes bound to genomic DNA (103, 104). They serve as gatekeepers by modulating access of DNA-binding transcription factors to the genome to regulate cell type-specific gene expression programs.

Additionally, certain processes directly modify chromatin components including histones and DNA. Molecular modifications to histones including methylation, acetylation, and ubiquitination serve as binding sites for cellular transcriptional machinery to up- or downregulate transcription of nearby genes (105–107). Mutations to histone modifiers or the histone proteins themselves can disrupt proper histone modification, leading to abnormal chromatin configurations (17, 108). Similarly, DNA methyltransferases (DNMTs) repress gene expression by direct methylation of cytosine residues of genomic DNA (109). The subsequent sections will explore how these chromatin modification mechanisms contribute to the development of pediatric brain tumors.

### 2.1 Chromatin remodelers

Chromatin remodelers are multiprotein complexes that utilize the energy of ATP hydrolysis to mobilize and restructure nucleosomes for gene regulation (103, 104). So far, four subfamilies of the ATP-dependent chromatin remodeling complexes have been identified: SWItch/Sucrose Non-Fermentable (SWI/SNF), Imitation SWItch (ISWI), Chromodomain Helicase DNA binding protein (CHD) and INOsitol 80 (INO80) subfamilies (11, 104). In pediatric brain tumors, abnormal alterations in chromatin structure suppress neuronal differentiation programs, enhancing susceptibility of transformation into malignant cells by maintaining cell proliferation signals (10, 27, 37, 110).

**Figure 2:**
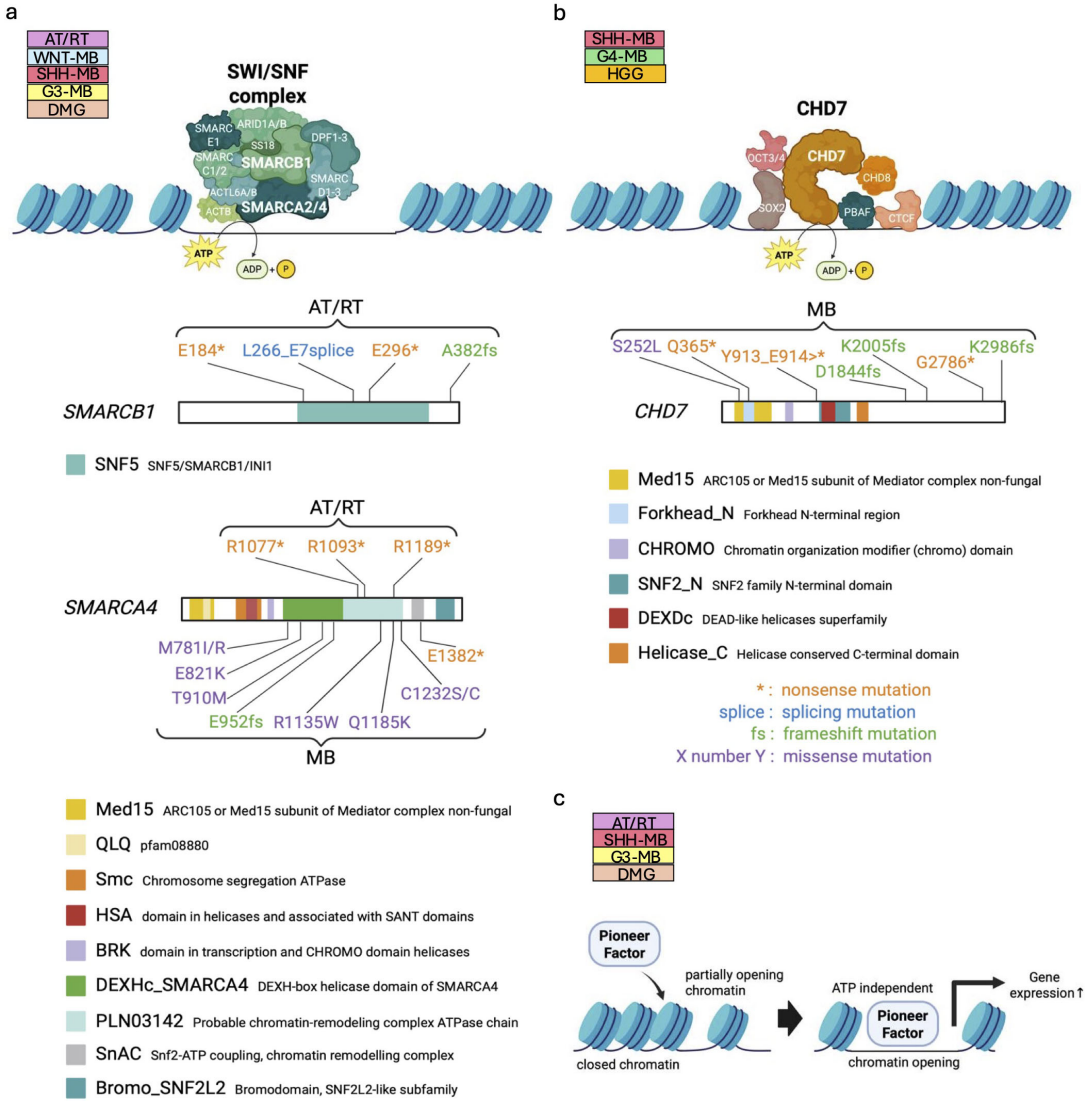
Dysregulation of chromatin remodelers in pediatric brain tumors. **(a, b)** Structure of ATP-dependent chromatin remodelers, the SWI/SNF complex (upper panel in a) and CHD7 (upper panel in **(b)**). The core subunits of the SWI/SNF complex, *SMARCB1* and *SMARCA4* are mutated in human patients bearing AT/RTs and MBs (111) (lower panels in **(a)**), while mutations of *CHD7* are found in MBs (38) (lower panel in **(b)**). The known functional domains of the respective proteins are highlighted and labeled with their names. The patterns of genetic mutations are shown in the figure. Data sourced from previous studies (38, 111) and St. Jude Cloud Pediatric Brain Tumor Portal (https://pbtp.stjude.cloud). **(c)** Schematic diagram of regulation of chromatin compaction by ATP-independent pioneer factors.

The SWI/SNF complex is one of the frequently mutated chromatin remodelers in pediatric brain tumors (7, 111, 112). In normal cells, the SWI/SNF complex functions to modify chromatin structure by recruiting other proteins that add epigenetic marks, including histone acetylation and methylation, to achieve proper chromatin compaction (**Figure 2a**). Such chromatin modification affects gene expression patterns essential for normal neurodevelopmental processes including cell cycle regulation (11), which is reflected in cancer genome sequencing data that reveal various genetic alterations in individual components of the SWI/SNF complex (111–113). For example, somatic mutation-based biallelic inactivation of *SMARCB1* (**Figure 2a**), a core component of the SWI/SNF complex, is frequently observed in Atypical Teratoid/Rhabdoid Tumor (AT/RT) (approximately 98%) (28). In line with this, loss of *SMARCB1* expression between embryonic day 6 and 10, but not during any other developmental periods, induced AT/RT formation in genetically engineered mouse models (27). Besides, *SMARCB1*-deficient human induced pluripotent stem cells (iPSCs) gave rise to AT/RT-like tumors (29), implying that embryonic stem cell (ESC)-like signature plays a crucial role in driving the malignant characteristics of AT/RT. LOF mutations in *SMARCA4* (**Figure 2a**), another key component of the SWI/SNF complex, have also been identified in AT/RT. *SMARCB1* and, rarely, *SMARCA4* are mutated in a mutually exclusive manner (30). This implies that dysfunction of the SWI/SNF complex is a main cause of AT/RT formation, and even single mutations in one of the main components in the SWI/SNF complex are enough to affect the division and differentiation of brain cells.

LOF of the SWI/SNF complex often collaborates with oncogenic signaling for tumorigenesis. In the initial phase of medulloblastoma (MB), cooperative interaction between *SMARCA4* loss and *CTNNB1* mutation promotes proliferation of embryonic cerebellar ventricular zone cells in mice, resulting in Wingless (WNT)-MB formation (31). Similarly, combinatorial loss of *SMARCA4* with overexpression of *MYC* increases proliferation of cerebellar granule neuron precursors (GNPs), leading to Group 3 (G3)-MB formation in mice (32). In addition, SMARCA4 interacts with DNA topoisomerase II α to facilitate DNA decatenation and its loss is associated with anaphase bridges that often result in partial chromosome gain or loss as well as polyploidy, which may predispose aneuploidy in MB (114). Meanwhile, no MB formation is observed with the loss of either *SMARCA4* or *SMARCB1* alone in mice (33). Rather, *SMARCA4* deletion in the murine cerebellum inhibited Sonic hedgehog (SHH)-MB formation, as SMARCA4 is required for activation of SHH signaling (33, 34). Similarly, as found in adult glioblastomas (35), CRISPR-based LOF and pharmacologic inhibition of *SMARCA4* revealed that SMARCA4 is required to maintain characteristics of H3K27M-driven diffuse midline glioma (DMG) (36). Thus, the SWI/SNF complex does not always function independently in tumor formation; instead, it sometimes contributes to establishing an epigenetic landscape that facilitates tumorigenesis driven by other cancer signals, with its role varying depending on the tumor type.

Alongside the SWI/SNF complex, dysfunction of CHD subfamily chromatin remodelers is also associated with failure of neural cell differentiation. CHD7 plays a key role in maintaining euchromatin (**Figure 2b**) via recruiting DNA topoisomerase II β to target genes required for differentiation of cerebellar GNPs (10). In addition to the hypothesis that imbalance between GNP differentiation and proliferation could result in SHH-MB formation in cerebellum (115), the observation of LOF mutations in *CHD7* (38) (**Figure 2b**) in MB warrants further investigation of the oncogenic mechanisms of *CHD7* mutations. To date, evidence that LOF mutations in *CHD7* promote pediatric brain tumors been limited to SHH-MB (39) and Group 4 (G4)-MBs (37). However, further elucidation of differentiation mechanisms in normal cells, together with a deeper understanding of *CHD7* mutations in tumorigenesis, is expected to lead to the identification of new molecular targets.

Conversely, functional CHD7 is suspected to trigger tumorigenesis in gliomas. *CHD7* is highly expressed in gliomas, and *CHD7* overexpression increased proliferation and maintained the stemness of neural stem cells (NSCs) and neural precursor cells (NPCs) (40). This study also revealed that silencing *CHD7* diminished the proliferation of glioma initiating cells, suggesting that CHD7 could be a potential therapeutic target of gliomas.

As shown above, the SWI/SNF and CHD chromatin remodeler complexes are involved in formation of some pediatric brain tumors. In contrast, little is known about the possible roles of the ISWI and INO80 complexes in pediatric brain tumors, and reports of their mutation are exceedingly rare. This could be explained by the possibility that the functions of these chromatin regulators are compensated by other molecules in their cells of origin, or that these regulators are crucial for the survival of these cells. These intriguing possibilities warrant further investigation, and we look forward to future research shedding light on them.

Pioneer factors (PFs) are known not only to function as transcription factors but also to modify chromatin structure independently of ATP. Mechanistically, PFs directly recognize nucleosome motifs, bind to closed chromatin, and alter DNA accessibility, thus enabling the reprogramming of cell fate decisions (44) (**Figure 2c**). Among the many known pioneer factors, ASCL1 (116), NEUROD1 (117, 118), OTX2 (42), SOX2 (45, 119, 120), and SOX9 (48) play crucial roles in pediatric brain tumor formation as well as tissue-specific chromatin regulation and are also involved in a range of neural developmental processes. These PFs are also closely associated with pediatric brain tumor formation. In cellular models, chromatin modifications mediated by ASCL1 and NEUROD1 are implicated in transcriptional circuitry of H3.3K27M-driven DMG (41) (see section 1.3). In G3-MB, genome-wide chromatin and expression profiling have shown that NEUROD1 acts as a key transcriptional mediator for tumor growth. NEUROD1 cooperates with another pioneer factor, OTX2, to shape the regulatory landscape of G3-MB through cooperative activity at enhancer elements and promotes the expression of target genes (42). Conversely, in SHH-MB, mouse models have demonstrated that *NEUROD1* overexpression enhances differentiation of tumor cells and inhibits tumor growth (43). These findings suggest that NEUROD1 has different functions depending on the tumor type as also seen in other chromatin remodelers. Similarly, SOX9 also exhibits distinct epigenomic regulation across different tumor types. SOX9 suppresses high grade glioma (HGG) growth and expands acetylation of histone H3 at lysine 27 (H3K27ac), but facilitates zinc finger translocation-associated (ZFTA) fusion-positive supratentorial ependymoma (ST-EPN-ZFTA) development by altering H3K27ac occupancy (48). These tumor-type-specific function of pioneer factors may reflect differences in epigenomic landscapes of cellular origins of each tumor. In addition to these factors, SOX2 is also a crucial component of the transcriptional circuitry in some kinds of brain tumor such as DMG (44, 46), AT/RT (44, 47), and SHH-MB (45), and maintains neural stemness and developmental potency in tumor cells. However, it remains unknown what specific chromatin changes SOX2 induces during tumorigenesis.

Nuclear Factor I (NFI) family proteins not only regulate gene expression as a transcription factor but also maintain open chromatin architecture by binding to open chromatin regions, albeit little evidence as PFs (121). NFIB, a member of the NFI family, plays an important role in brain development, including cerebellar formation and neuronal migration (122–124). Along with its role in normal brain development, we have recently discovered that SHH-MB-specific NFI-binding open chromatin regions emerge in precancerous GNPs, then NFIB binds to these open chromatin regions and maintains the chromatin structure. These chromatin alterations mainly occur at the transition from GNPs to hyperplasia during SHH-MB progression, then strengthening oncogenic pathways including the SHH signaling pathway (49). Thus, ATP-independent chromatin modulators are also emerging as an essential factor for pediatric brain tumor formation, although there is still much unclear about functions in detail.

Aside from transcriptional activators as discussed above, repressive chromatin regulators also play a role in neurodevelopment and cancer. For example, repressor element 1 silencing transcription factor (REST) serves as a key regulatory factor by repressing the transcription of genes involved in neuronal differentiation and maturation. REST forms a complex with the REST corepressor (CoREST), and it recruits chromatin-modifying enzymes to induce a condensed chromatin state. CoREST functions not only as a corepressor but also plays distinct roles in neuronal differentiation and maturation independently of REST (125). During normal development, the CoREST complexes comprising SMARCA4, lysine-specific histone demethylase 1A (LSD1) and histone deacetylase 1/2 (HDAC1/2) methyl and acetyl groups from histone H3 at lysine 4 (H3K4) and lysine 9 (H3K9), respectively. These modifications facilitate chromatin condensation through the recruitment of heterochromatin protein 1 (HP1) and methyl CpG-binding protein 2 (MeCP2), thus leading to transcriptional silencing of target genes (125–127). In the context of pediatric brain tumor formation, the CoREST complex is degraded in G3/G4 MBs due to *KBTBD4* mutations, which impair its E3 ubiquitin ligase function. Consequently, CoREST target genes, including those involved in stemness, become aberrantly activated, ultimately promoting G3/G4 MB tumor growth *in vitro* (50). In addition, CoREST and the NuRD chromatin remodeling complex have been shown to contribute to EGFR silencing and may function as tumor suppressors in the breast cancer cells (128–130). Thus, dysregulation of repressive chromatin remodelers may not only disrupt normal neurodevelopmental trajectories but also contribute to oncogenic transformation in pediatric brain tumors.

### 2.2 Histone modifiers

Another well-studied epigenetic gene regulatory mechanism for chromatin remodeling is direct modification of the histones. Histones are octamer complexes composed of two subunits each of H2A, H2B, H3, and H4. Histone modifiers regulate gene expression through various modifications of histones. Methylation and acetylation primarily occur on H3 and H4, while ubiquitination mainly takes place on H2A and H2B.​​ Acetylation of H3K27 is associated with open chromatin, as weakening the binding of DNA to histones by adding a negatively charged carboxyl group to the lysine residue. Due to this, the genomic regions marked by H3K27ac are accessible for gene transcription and are defined as active enhancers of transcription. Histone acetyltransferases (HATs) catalyze such histone acetylation, while HDACs negatively regulate this modification, often resulting in gene silencing. Unlike histone acetylation, histone methylation occurs at lysine or arginine residues in the histone tail and is regulated by histone methyltransferases (HMTs), with gene transcription being either activated or repressed depending on the type of amino acid residue modified and the site of methylation. HMT-based modification comprises mono-, di-, and tri-methylation of histones, whereas histone demethylase facilitates the removal of methyl groups. Specifically, trimethylation of H3K4 (H3K4me3) are strongly associated with active promoters. On the other hand, H3K27me3 is enriched in heterochromatin and is linked to gene silencing. Mono-ubiquitination of histone H2A is also known to be responsible for gene silencing (105–107). Therefore, mutation or dysregulation of proteins responsible for histone modifications can disrupt cellular gene expression patterns, often contributing to cancer progression. So far, various studies have employed brain tumor models to explore which alterations or dysregulation of histone-modifying enzymes contribute to tumor development. This section introduces several notable histone modifiers and their roles in pediatric brain tumor development.

**Figure 3:**
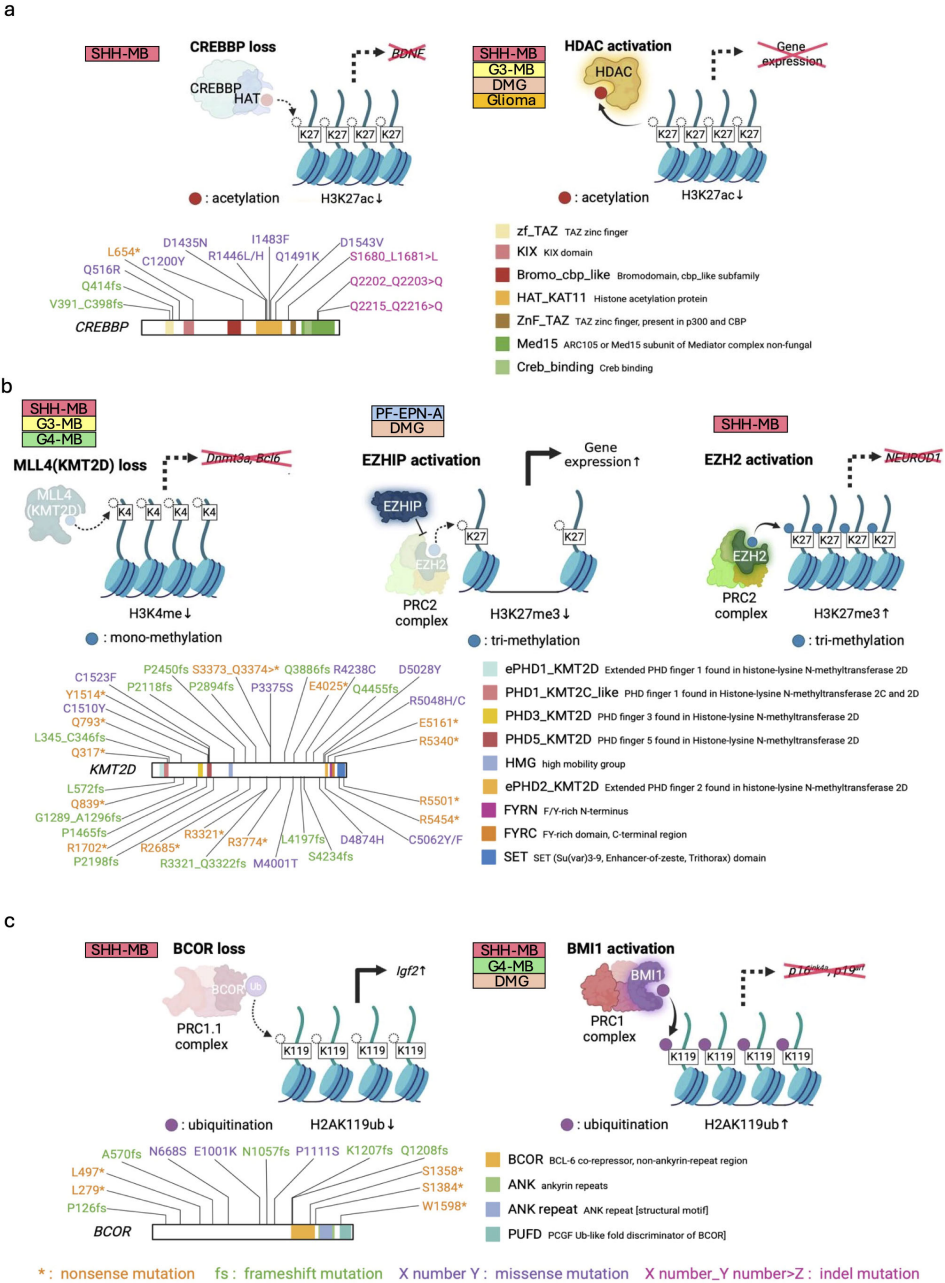
Failure of appropriate histone modification in pediatric brain tumors. **(a)** Inhibition of chromatin opening by loss of HAT function (e.g., CREBBP) (upper left panel) and aberrant activation of HDAC (upper right panel). Pathogenic loss-of-function (LOF) mutations of *CREBBP* in MB (38, 51) (lower panel). **(b)** Histone H3 demethylation by loss of KMT function (upper left panel) and EZHIP activation (upper middle panel) for tumorigenesis. Conversely, in some tumors, EZH2 activation often functions to prevent proper differentiation and contributes to oncogenesis (upper right panel). Mutations of *KMT2D* found in MBs (lower panel) (38). **(c)** Histone H2A ubiquitination regulating cancer-related genes. BCOR loss activates *Igf2* oncogene (upper left panel), while BMI activation inhibits tumor suppressor genes (upper right panel). LOF mutations of BCOR in MBs (68) (lower panel). The known functional domains of the respective proteins are highlighted and labeled with their names. The patterns of genetic mutations are shown in the figure. Data sourced from previous studies (38, 51, 68) and St. Jude Cloud Pediatric Brain Tumor Portal (https://pbtp.stjude.cloud).

Tumor initiation is sometimes induced by inactivation of differentiation-associated genes due to HAT dysfunction. For example, in SHH-MB, mutations in the HAT domain of CREB-binding protein (CREBBP) downregulate brain-derived neurotrophic factor (BDNF) (**Figure 3a**), in turn inhibiting proper migration of GNPs and retaining them in the germinal zone on the cerebellar surface known as a proliferative niche (131). Indeed, postnatal loss of *CREBBP* synergizes with activation of SHH signaling to accelerate SHH-MB growth (51). Of interest, HATs acetylate not only histones to regulate chromatin compaction, but also some proteins to directly modulate their functions. Many HATs such as CREBBP, E1A-associated protein p300 (p300), and p300/CREBBP associated factor (PCAF) activate p53 tumor suppressor functions by acetylation (132, 133). Since SHH-MBs display enhanced aggressiveness in *Trp53*-deficient background (134) and *CREBBP* or p300 loss (51, 133, 135, 136), epigenetic regulation-independent regulatory mechanisms may also need to be considered for this type of entity. Thus, HAT mutation promotes tumor formation by epigenetic and non-epigenetic mechanisms.

Similar to LOF mutations of HATs, HDAC activation also attenuates histone acetylation, resulting in gene silencing and tumorigenesis (137), although no reports of *HDAC* amplification have been reported in pediatric brain tumors. (**Figure 3a**). In MYC-amplified G3-MBs, HDAC2 and MYC are co-bound in H3K27ac open chromatin regions, adjusting expression of MYC-dependent genes via histone deacetylation (52, 53). Moreover, as predicted from the functions of HAT described above, HDACs also directly deacetylate and activate proliferation-related molecules, such as GLI1 and GLI2 in SHH-MB formation (54, 55). Besides MB, isocitrate dehydrogenase (IDH)-mutant gliomas, occasionally observed in adolescent and young adult (138), exhibit upregulation of genes associated with HDAC activity (56) and show significant anti-tumor responses upon knock-down of *HDAC1* and *HDAC6* (57). Furthermore, HDAC is also associated with the H3K27M DMG in several preclinical models (58–60). Thus, HDACs contribute to the maintenance of cancer cells through epigenetic mechanisms, across various cancer types.

In addition to dysregulation of histone acetylation, mutations in some HMTs also promote brain tumor formation. Specifically, H3K4 methyltransferase Mixed-lineage leukemia 4 (Mll4) in mice, also known as Lysine Methyltransferase 2D (KMT2D) in human, regulates neuronal differentiation and tumor suppression, and its loss triggers G3-like MB development with upregulating oncogenic Ras and Notch pathways, and downregulating tumor suppressor genes (e.g., *Dnmt3a*, *Bcl6*) (61) (**Figure 3b**). Another investigation of SHH-MB mouse models has shown that heterozygous *Kmt2d* loss, combined with abnormal SHH pathway activation, increases hindbrain invasion and spinal cord metastasis through downregulation of differentiation-associated and tumor suppressor genes, and upregulation of progression- and metastasis-related pathways/genes (e.g., TGFβ-signaling, NOTCH-signaling, *Atoh1*, *Sox2*, and *Myc*) (62). Notably, *KDM6A, KMT2C*, and *KMT2D* mutations also frequently occur in human SHH- and G4-MBs (38). Given that these molecules form the core nuclear regulatory structure, so-called the COMPASS complex (139), how deficiency of the COMPASS complex function regulates MB formation remains to be investigated.

Another HMT pivotal in brain tumor formation is Enhancer of Zeste Homolog 2 (EZH2), a key component of Polycomb Repressive Complex 2 (PRC2). EZH2 drives chromatin silencing by catalyzing the trimethylation of H3K27 (H3K27me3). Conversely, EZH Inhibitory Protein (EZHIP) disrupts this process by inhibiting EZH2 (**Figure 3b**). Alterations in H3K27me3 are linked to posterior fossa A ependymoma (PF-EPN-A), the most aggressive EPN subgroup, and DMG. PF-EPN-A exhibits global reduction in H3K27me3 alongside elevated *EZHIP* expression (63, 64). Previous studies demonstrated that *EZHIP* knockout inhibits PF-EPN-A cell growth *in vitro* (65). Although further *in vivo* validation using animal models is still required, the high expression of *EZHIP* and the resulting H3K27me3 reduction might be potential drivers of PF-EPN-A tumorigenesis. In DMGs, the hallmark mutation of H3K27M inhibits H3K27 histone trimethylation by PRC2 (see section 1.3). This mutation mimics the function of EZHIP, and in some cases of DMGs, elevated EZHIP levels have been reported as an alternative to the H3K27M mutation (66). Of interest, recent study has demonstrated that such common molecular features between PF-EPN-A and DMG are linked closely to expression patterns of genes (e.g., *CRABP1*) in human hindbrain development (140). Whether the failure of EZH2/EZHIP-mediated epigenetic histone regulation mimics an epigenetic state normally restricted to the developing hindbrain for their tumorigenesis remains to be further studied.

Aside from H3K27me3 loss, H3K27me3-based gene repression is inversely implicated in other types of brain tumors. In IDH-mutant gliomas, IDH mutations promote histone methylation marks including H3K27me3 by epigenetic reprogramming (141) Meanwhile, in SHH-MB, EZH2-mediated H3K27me3 marks accumulate at *NEUROD1* regulatory elements, suppressing *NEUROD1* expression and maintaining tumor cells in an undifferentiated state (**Figure 3b**). Consistently, pharmacological inhibition of EZH2 reduces H3K27me3, upregulates *NEUROD1*, and drives tumor cell differentiation, thereby inhibiting tumor growth (43). Remarkably, once SHH-MB cells are differentiated through such mechanisms, they permanently lose their proliferative ability and tumorigenic functions (43). Another study showed that complete deletion of PRC2 reduced occurrence of SHH-MB because PRC2 is required for maintenance of GNPs, but partial deletion of PRC2 led to SHH-MB growth through increased expression of oncogenes such as *Igf2* and non-cell autonomous mechanism with paracrine IGF2 signaling (142). Additionally, H3K27me3 demethylase UTX/KDM6 plays crucial roles in GNP differentiation through *NEUROD2* expression and recruits immune cells to the tumor microenvironment, thereby UTX/KDM6A deletion contributes to SHH-MB development by maintaining undifferentiated and immunologically cold states (67). Overall, epigenetic modification changes of H3K27me3 by dysregulation of EZH2/EZHIP and/or histone demethylases are intimately involved in pediatric brain tumors.

The gene silencing mechanism by histone H2A mono-ubiquitination is also closely associated with cancer. Both canonical PRC1 and non-canonical PRC1.1 ubiquitinate histone H2AK119 via RING1A/B, an E3 ligase within the complex (**Figure 3c**). Canonical PRC1 represses expression of tumor suppressor genes (e.g., *p16^Ink4a^*, *p19^Arf^*) through histone ubiquitination (143) and *BMI1*, a core component of PRC1, is upregulated in various types of cancers (144). Hyper-physiological expression of *BMI1* downregulates tumor suppressor genes and disrupts normal developmental signaling in the cerebellum (**Figure 3c**), thereby contributing to SHH-MB tumorigenesis (69). In xenograft models of G4-MB, *BMI1* knockdown suppress their tumor growth and invasion (37, 70). Besides MBs, H3K27M DMGs also upregulate *BMI1* and are susceptible to its inhibition (71). In contrast, LOF mutations in non-canonical PRC1.1 components occasionally activate oncogenes for pediatric brain tumor formation. For example, our previous study demonstrated that LOF mutations in BCL6-Co-Repressor (BCOR), a component of PRC1.1, enhance the aggressiveness of SHH-MBs via upregulation of Insulin-like growth factor 2 (Igf2), a strong mitogen for GNPs, due to failure in gene silencing mediated by histone ubiquitination (68). (**Figure 3c**). As with other histone modifications, understanding of which genes are regulated by histone ubiquitination is crucial for the phenotype of tumor cells.

Notably, a recent study has further identified that neurotransmitters such as serotonin also modify histones. Mouse models of ST-EPN-ZFTA have demonstrated that serotonin secreted from serotonergic neurons is transported into the nucleus of tumor cells, in turn modifying histones. This histone serotonylation promotes the expression of *Etv5* by opening chromatin. The elevated ETV5 then transcriptionally represses the tumor suppressor neuropeptide Y (NPY), facilitating EPN tumor formation (145). Such an entirely new mechanism of histone modification by neurotransmitters is now gradually becoming clear from the cancer neuroscience field and could pave the way for novel therapeutic avenues.

### 2.3 Histone mutations

Histone modifications play a vital role in the precise regulation of gene expression. Mutations at these modification sites can disrupt cellular gene expression networks, sometimes driving tumorigenesis. Pediatric brain tumors are no exception, with some histone mutations reported. Indeed, pathogenic histone mutations are largely restricted to H3K27 and H3G34, which are linked to DMGs and pediatric HGGs (pHGGs). In recent years, multifaceted research approaches are advancing our understanding of the oncogenic signaling pathways triggered by these mutations. The following section describes, based on the latest knowledge, how histone mutations influence the formation and growth of pediatric brain tumors.

**Figure 4:**
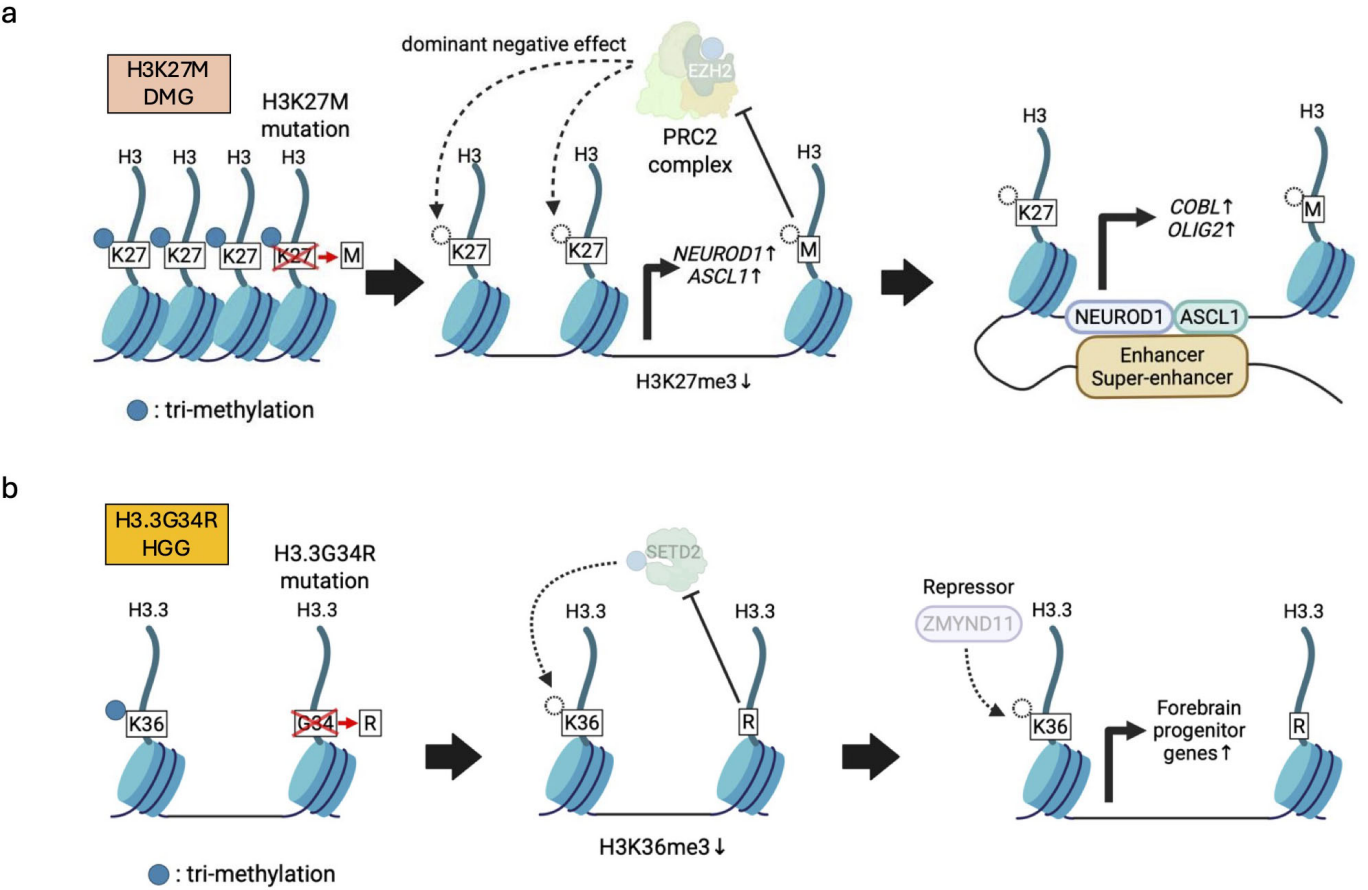
Histone mutations and the resulting abnormalities in histone modifications. **(a)** H3K27M-mutant H3 exhibits higher binding affinity than wild-type histone H3 to PRC2, leading to global reduction of PRC2-mediated H3K27me3 (left). Decreased H3K27me3 induces expression of pioneer factors (e.g., *NEUROD1*, *ASCL1*) (middle) that subsequently cooperate with enhancer/super-enhancers to further enhance abnormal chromatin accessibility (right). **(b)** H3.3G34R represses a lysine 36 methyltransferase SETD2, leading to low H3.3K36me3 levels, which disrupts interaction between a repressor ZMYND11 and H3.3. Reduced ZMYND11 function, in turn, activates forebrain progenitor genes involved in hemispheric pHGG formation.

H3K27M, missense mutation in histone H3 at amino acid 27, lysine (K) to methionine (M), is one of the best-studied histone mutations in pediatric brain tumors (**Figure 4a**). H3K27M mutations are seen in about 80% of DMG. Approximately 75% of H3K27M mutations are found in *H3-3A* (*H3F3A*) encoding H3.3, while the remaining 25% are seen in *H3C2* (*HIST1H3B*) encoding H3.1 (17, 22). Although H3K27M mutations occur only in 5-17% of the total H3 expressed (17), they potentially have a dominant negative effect to inhibit PRC2, resulting in global loss of H3K27me3 and enhanced gene expression in those loci. This can be explained by the mechanism through which H3K27M exhibits higher binding affinity to PRC2 than wild-type H3K27 and reduces EZH2 auto-methylation, further limiting the methyltransferase activity of PRC2 (17, 72). However, H3K27me3 are still retained in several genes by residual PRC2, and DMGs also require PRC2 for proliferation. Thus, inhibition of PRC2 could be a potential therapeutic strategy for DMG patients (146). The H3K27M mutation-induced H3K27me3 reduction causes chromatin structure opening, leading to increased expression of PFs, such as NEUROD1 and ASCL1. This processes subsequently enhance chromatin accessibility and upregulate the expression of neurogenesis- and oncogenesis-related genes (e.g., *COBL*, *OLIG2*), thereby triggering tumorigenesis (41) (**Figure 4a**). In line with this, the H3K27M mutation disrupts normal differentiation, while promoting proliferation and stemness phenotypes in human ESCs (73) as well as human fetal NSC models (74). Even so, H3K27M alone is insufficient for tumorigenesis. In human fetal NSC models, H3.3K27M enhanced clonogenicity and reduced senescence only in the brainstem but not in the forebrain, implying that regionally specific developmental cellular characteristics and their microenvironment could be required (74). It has also been demonstrated that H3K27M in combination with PDGF signaling activation and *TP53* knockdown causes tumorigenesis from hES-derived NSCs, NPCs (75–77), and OPCs (76). In these genetic backgrounds, the oncogenic effects of H3K27M depend on the cell of origin, with H3K27M being more tumorigenic in NSCs or NPCs than in OPCs (75–77). Our previous research using the models derived from human iPSCs demonstrated that induction of H3.3K27M and *TP53* inactivation gave rise to DMG only from NSCs but not from OPCs (78). Collectively, the H3.3K27M-driven epigenetic state seems to collaborate with region-specific and cell-type specific epigenetic programs for transformation into DMGs.

H3G34R/V is another remarkable histone mutation found in pediatric glioma. H3G34R/V indicates missense mutations in histone H3 at amino acid 34, glycine (G) to arginine (R) or valine (V). H3G34R/V mutations are identified exclusively in *H3-3A* (*H3F3A*) encoding H3.3 (17) (**Figure 4b**). More than 30% of pHGGs arising in the cerebral hemispheres contain H3.3G34R/V mutations (79, 80). Although the function of H3.3G34R/V mutations are not fully understood, hESC models have shown that H3.3G34R along with knockout of *ATRX* and *TP53* blocked differentiation and enhanced proliferation of tumor cells resembling interneuron progenitor cells in the ventral forebrain, but had no effect in ventral hindbrain spheroids (81). This suggests that H3.3G34R also confers a selective oncogenic advantage in specific regions. Consistent with this idea, H3.3G34R/V pHGGs arise in hemispheric or cortical regions, while H3K27M DMGs are found in pontine or supratentorial midline regions (17). At the molecular level, H3.3G34R, *ATRX*, and *TP53* mutations cooperate to affect RNA splicing through suppression of intron retention, leading to increased expression of *NOTCH2NL* and eventually promoting tumor growth and survival (81). Furthermore, H3.3G34R mutations cause the loss of adjacent H3.3K36me3 by inhibiting SETD2, a lysine 36 methyltransferase, at the promoters and genetic regions of forebrain-associated genes, and disrupt interactions between H3.3 and ZMYND11, a transcriptional repressor that specifically reads H3.3K36me3. As a result, the failure of ZMYND11 possibly enhances the expression of forebrain progenitor genes (74) (**Figure 4b**). Thus, H3.3G34R/V is involved in the pathogenesis of hemispheric pHGGs in a different manner from H3.3K27M.

### 2.4 DNA methylation

Histone modifications, as mentioned above, regulate transcription from genomic DNA either positively or negatively. Direct modifications of genomic DNA itself also affect gene transcription. DNA methylation is a well-known molecular machinery that negatively regulates transcription. Distinct DNA methylation regions are characteristic of each cancer type, likely reflecting the identity of the tumors themselves or the cells of origin, and diagnostic methods utilizing these patterns (147) have become increasingly popular.

**Figure 5:**
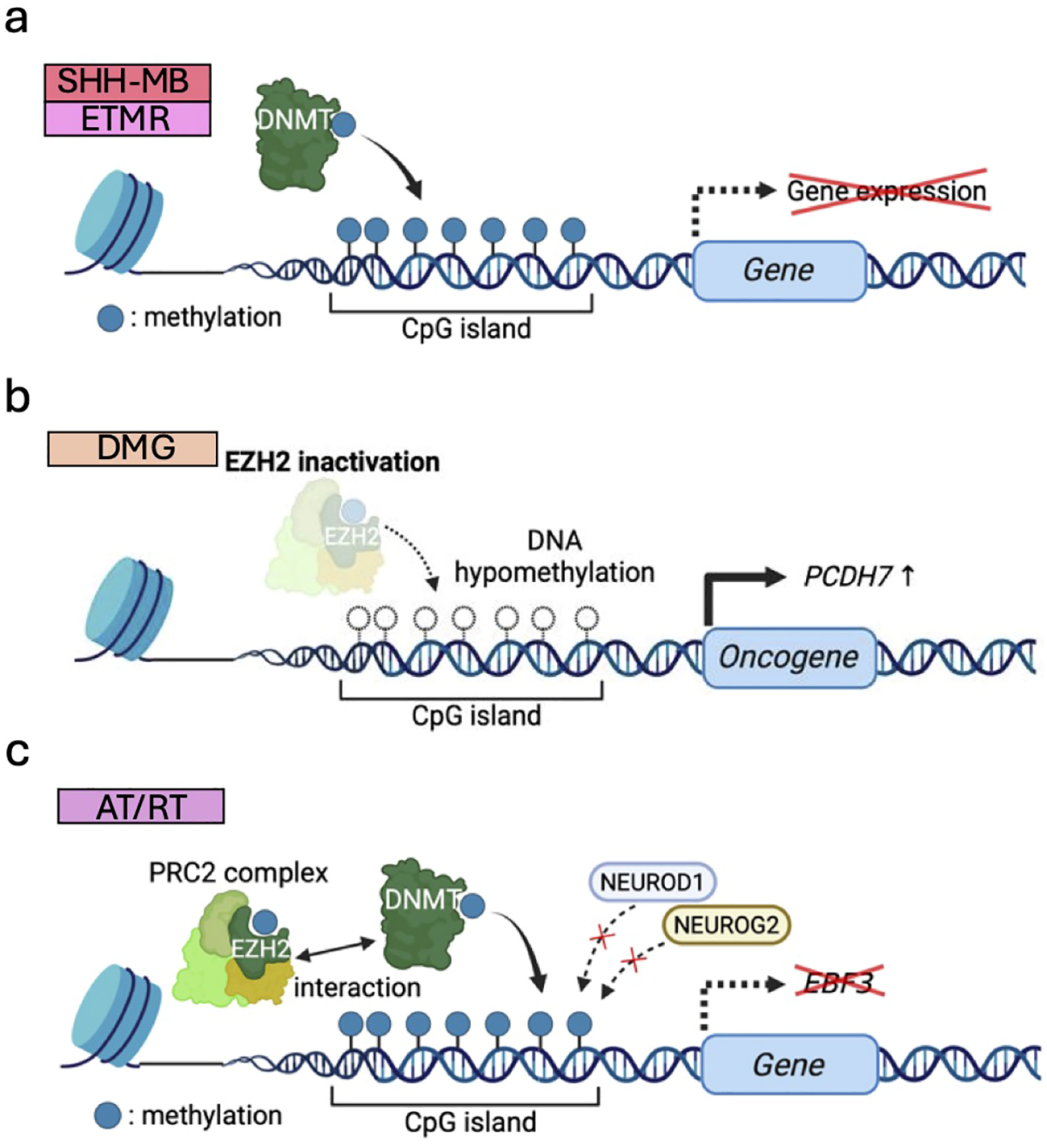
DNA methylation dysregulation associated with pediatric brain tumor formation. **(a)** Upregulated DNMTs methylate CpG islands of genomic DNA and silence transcription in SHH-MB and ETMR. **(b)** Global DNA hypomethylation due to EZH2 inactivation induces oncogene expression (e.g., *PCDH7*) to drive DMG tumorigenesis. **(c)** DNA hypermethylation caused by EZH2 and DNMTs blocks the binding of transcription factors (e.g., NEUROG2/NEUROD1), repressing differentiation-associated genes (e.g., *EBF3*) and maintaining stemness of AT/RTs.

DNMTs add methyl groups to cytosine bases in genomic DNA, altering transcription factor binding kinetics and recruiting transcriptional repressor complexes such as PRC2 to silence transcription (109). Recently, genome-wide CRISPR-Cas9 knockout screens in murine MB models illustrated that DNMTs are vital for normal murine cerebellar development and required for SHH-MB tumorigenesis (82) (**Figure 5a**). Pharmacological inhibition of DNMT1 reduced tumor growth in a cell line and *in vivo* mouse models of SHH-MB (82). Furthermore, *TTYH1*::*C19MC*-driven abnormal activation of *DNMT3B* is a hallmark of embryonal tumors with multilayered rosettes (ETMRs) and accounts for 90% of ETMR tumors (83, 84). In preclinical settings, DNMT inhibitors have been shown to suppress the growth of ETMR cell lines by inducing cell death and differentiation (85). As such, the role of DNMTs in tumorigenesis and their potential as therapeutic targets are increasingly gaining attention.

DNMT-mediated DNA methylation has been reported to be modified by EZH2 through their direct interaction with each other (148). In DMG, reduced H3K27me3 and DNA hypomethylation, possibly due to failure of proper EZH2 recruitment, cooperate to activate ectopic gene expression (e.g., *PCDH7*) and drive tumorigenesis (72) (**Figure 5b**). It is suggested that decreased levels of H3K27me3 by H3.3K27M mutation may predispose to global DNA hypomethylation (72). This may be explained by the mechanisms in which H3K27M exhibits higher binding affinity to PRC2 than wild-type H3K27, thereby limiting the methyltransferase activity of PRC2 and resulting in global DNA hypomethylation (17). In AT/RT, DNA hypermethylation is frequently observed partially due to DNMT1 and DNMT3A upregulation, and DNMT inhibitors impaired tumor growth *in vitro* and *in vivo* (149). EZH2 also interacts with DNMTs and promotes DNA hypermethylation, leading to suppression of neural differentiation factors like NEUROG2/NEUROD1. This downregulates neural differentiation-associated genes (e.g., *EBF3*) and maintains PSC-like DNA methylation and gene expression patterns specific to this type of tumor (86) (**Figure 5c**). Accordingly, EZH2-mediated DNA methylation at specific sites is closely related with tumorigenesis.

## 3 Genomic rearrangement

Genomic rearrangement is one of the characteristic phenomena often caused by erroneous double strand break repair, chromosomal segregation failures, and chromothripsis (150–152), and it can sometimes contribute to tumor formation by inducing abnormal gene expression. Recent advancements in understanding epigenome-regulated gene expression have increasingly elucidated novel cancer epigenomic mechanisms arising from genomic rearrangements (152, 153). Genomic rearrangement can modify chromatin topology, including chromatin looping and distal chromatin interactions, links oncogenes to distal transcriptional regulatory elements called enhancers. Structural rearrangement may allow oncogenes to utilize enhancers of other genes, a phenomenon called enhancer hijacking. In addition, oncoproteins or microRNA (miRNA) from fusion genes occasionally caused by genomic rearrangement have been revealed to epigenetically regulate tumor development. As another finding, genomic rearrangement can generate extra chromosomal DNA (ecDNA), circular chromatin that exists outside chromosome structures within the cell nucleus and plays a crucial role in shaping cancer epigenome. This chapter focuses on cancer signaling pathways linked to epigenomic changes that stem from such genomic rearrangement.

### 3.1 Enhancer hijacking

**Figure 6:**
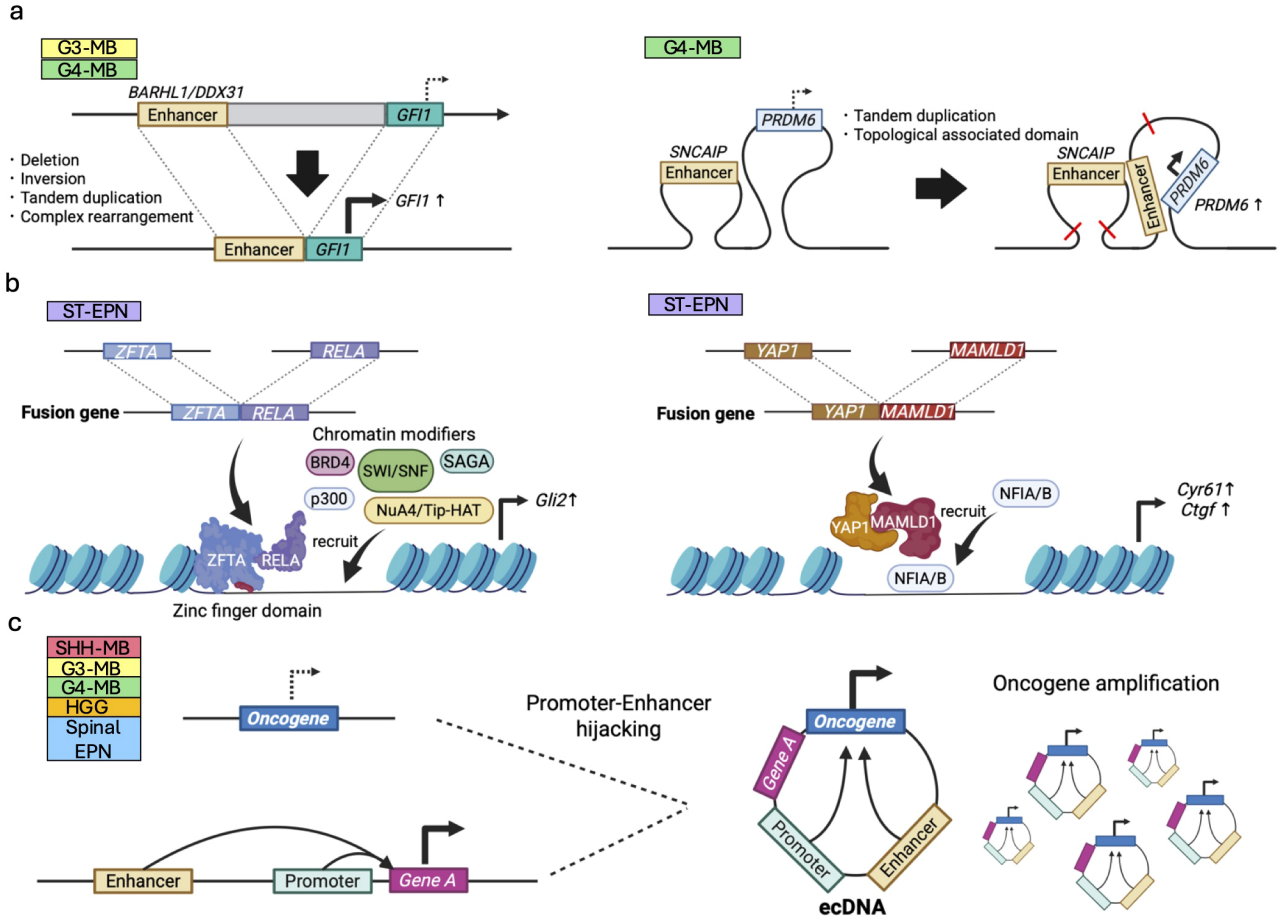
Epigenetic dysregulation induced by genomic rearrangement in pediatric brain tumors. **(a)** Enhancer hijacking; oncogenes (e.g., *GFI1*) are aberrantly activated by enhancers of other genes (e.g., *BARHL1/DDX31*) via deletion, inversion, tandem duplication due to genomic rearrangement in G3/G4-MBs (left panel). Topological associated domains and chromatin looping can also lead to the enhancer hijacking (right panel). **(b)** Fusion oncoproteins; ZFTA::RELA (left panel) and YAP1::MAMLD1 (right panel) observed in ST-EPNs expressed from these fusion genes modulate chromatin states in combination with other chromatin regulators. **(c)** Structural rearrangements leading to ecDNA formation may juxtapose oncogenes with ectopic enhancer elements, leading to transcriptional dysregulation of the ecDNA-amplified oncogene.

Enhancer hijacking events are caused by abnormal genomic rearrangements and chromatin configurations, where cancer-related genes are aberrantly activated by enhancers that are originally designated for other genes. For instance, enhancers of *BARHL1*/*DDX31* activate *GFI1/GFI1B* transcription following genomic rearrangements for G3/G4-MB growth (87) (**Figure 6a**). A follow-up study has revealed physical interaction between GFI1 and LSD1 for tumorigenesis, identifying an LSD1 inhibitor as a new potential therapeutic drug for GFI1/MYC-driven MB (88) Thus, understanding of the mechanisms underlying abnormal enhancer activities within cancer genomes leads to the discovery of novel therapeutic targets.

Following this line of research, extensive efforts have been made to elucidate enhancer regions in various pediatric brain tumors. For instance, some G4-MBs upregulate *PRDM6* expression by SNCAIP-mediated G4-specific enhancer hijacking (38) (**Figure 6a**). Consistent with the fact that *PRDM6* is known to be a histone methyltransferase, the latest study has demonstrated that PRDM6 binds to H3K27me3 and exerts widespread repression of chromatin accessibility. Overexpression of *PRDM6* alone in iPSC-derived neuroepithelial stem cells led to tumor formation albeit with molecular characteristics resembling those of G3-MBs (89), highlighting the oncogenic potential of *PRDM6* in a human genetic background.

In addition, recent high-throughput chromosome conformation capture (Hi-C) analyses on supratentorial ependymoma with ZFTA fusion (ST-EPN-ZFTA) and PF-EPN-A have found that 3D genome conformation activates the genes essential for their survival through enhancer hijacking, thus often causing cancer type-specific vulnerabilities (90). In pHGGs and DMGs, structural variants (SVs) drive MYC activation primarily through enhancer hijacking rather than gene amplification. Regulatory elements near *PVT1* and *CCDC26* are frequently co-opted, leading to aberrant MYC overexpression and highlighting enhancer reorganization as a key mechanism of tumorigenesis (91). Another recent study showed that somatic SVs enriched for enhancer hijacking also play a major role in shaping the cancer DNA methylome and regulating the expression of nearby genes, such as *MYC*, *MYCN*, *TERT*, *ZFTA*, *KIAA1549*, *ATRX*, and *CDKN2A*, in pediatric brain tumors (154).

Of note, enrichment of the cells carrying such enhancer hijacking events within cancer may provide some clues about the cellular origins of cancer. If this event is important for tumor initiation, the abnormally used enhancers could be active in their cellular origins (155). An *in vivo* reporter assay in mice (156, 157) would be a powerful tool to identify the cell of origin by analyzing the activity of the identified enhancers in the developing brain.

### 3.2 Fusion genes

Another epigenomic regulatory mechanism resulting from genomic rearrangements is the formation of cancer-specific fusion genes by combining two previously separate genes. The resulting abnormal fusion proteins or miRNA can function as oncogenic molecules for the development and progression of brain tumors. While a wide range of fusion genes have been identified in pediatric brain tumors (97), we highlight here those that are particularly involved in epigenetic regulation.

Unlike enhancer hijacking events, this type of mechanism regulates cancer epigenomes indirectly. *ZFTA* fusion genes, recently identified in ST-EPNs, are composed of a segment of the *ZFTA* gene fused with various transcription activators including *RELA*. ZFTA fusions have been reported to have an oncogenic capacity *in vivo* using animal models and to drive epigenetic changes and activate downstream oncogenic transcription programs, including *Gli2* activation (92, 93). The portion of ZFTA plays a crucial role in chromatin binding and remodeling via its zinc finger DNA-binding domains, as well as its translocation into the nucleus (94). This fusion interacts other chromatin modifiers such as SWI/SNF, Spt-Ada-Grn5 acetyltransferase (SAGA) and NuA4/Tip60 histone acetyltransferase (NuA4/Tip-HAT) (94) (**Figure 6b**), hypothesizing its contribution to profound chromatin landscape modification, in turn converting various genes to a transcriptionally active state.

Another commonly detected fusion gene in ST-EPNs involves the activity of Yes-associated protein 1 (YAP1), a component of the Hippo signaling pathway that promotes cell growth and prevents cell death. Our previous study has shown that the segment of its primary fusion partner, MAMLD1, directs YAP1::MAMLD1 to the cell nucleus and attracts chromatin modifiers NFIA/B to specific loci on YAP1 target genes, such as *Cyr61* and *Ctgf* (**Figure 6b**). This process amplifies the cancer-promoting YAP1 signaling pathway (95). Among the various fusion genes, CIC fusions are observed in certain types of pediatric brain tumors, such as *CIC::NUTM1* in CNS Ewing sarcoma family tumors with CIC alterations (CNS EFT-CIC) (96) and *CIC::LEUTX* in anaplastic pleomorphic astrocytomas and CNS embryonal tumors (97). Of note, *CIC::DUX4* fusions, which are detected in Ewing sarcomas but not in CNS tumors, have recently been revealed to function as transcriptional activators and to modify chromatin states through direct interaction with the acetyltransferase p300 (158). Such chromatin regulatory mechanisms driven by CIC fusions may also be involved in CNS tumors.

As another example, tumorigenesis of ETMRs also seems to be triggered by specific fusion genes. ETMRs are characterized by the miRNA cluster amplification caused by recurrent gene fusions of chromosome 19 miRNA cluster (C19MC) and tweety family member 1 chloride ion channel (*TTYH1*). The *TTYH1*::*C19MC* fusion structure enhances the expression of C19MC miRNAs, which downregulate the transcriptional repressor *RBL2* and in turn, upregulate *DNMT3B*, leading to ETMR development as mentioned above (84). Collectively, cancer-specific fusion genes not only regulate molecules involved in cell proliferation and survival but also possess functions that create a cancer-supportive epigenome conducive to cellular growth. They achieve this by interacting with various epigenomic factors to establish an environment favorable for tumor progression.

In addition to the fusion genes presented in this study, some other fusion genes, such as those involving BCOR, p300 and BEND2, may also contribute to the cancer epigenome, given that their original functions are related to chromatin modifications (97, 159, 160). However, further studies are needed to elucidate the mechanisms by which these fusion genes promote tumor progression.

### 3.3 Extrachromosomal DNA

Genomic rearrangements in cancer occasionally produce circular chromatin, lacking centromeric sequences and usually 50kbp to 10Mbp in length, called extrachromosomal DNA (ecDNA) or double minutes (dm). Replication and segregation of ecDNA is decoupled from that of chromosomal DNA, enabling a tumor to accumulate high-copy amplification of ecDNA under positive selection (161). In human cancers, ecDNA is believed to drive malignant tumors by various mechanisms including amplification and overexpression of oncogenic sequences (162) including fusion oncogenes (163, 164), tumor evolution (161), chromatin remodeling (165), enhancer hijacking (166–168), and promoter hijacking (169, 170) (**Figure 6c**). Here we briefly review the role of ecDNA in epigenetic dysregulation of pediatric brain tumors and refer the reader to a recent review of transcriptional regulation by ecDNA across human cancers (171).

ecDNA in pediatric brain tumors has been reported in MBs (98, 99), pHGGs (91, 100, 101), and spinal EPNs (102). *MYCN* was most frequently amplified on ecDNA in MBs and spinal EPNs, although in MB ecDNA amplifications may alternately target epigenetic regulators including *SETBP1* and *KMT2E* (98). To our knowledge, no ecDNA amplifications of *ZFTA* nor *YAP* fusion genes in EPNs have been reported to date.

In addition to oncogene amplification, the genomic rearrangements which produce ecDNA may also result in enhancer hijacking. In a small cohort of eight MB patient tumors profiled by Hi-C, half showed evidence of enhancer hijacking events between genomic loci from distal locations on the reference genome but juxtaposed on the ecDNA sequence due to genomic rearrangement (98). The assays required to detect regulatory interactions and ecDNA are not yet part of the standard of care for pediatric brain tumors. Thus, in our view, these observations probably represent an incomplete sample of the oncogenic amplifications and enhancer hijacking events which occur on MB and other pediatric brain tumors. We anticipate that future work will further illuminate the frequency and diversity of epigenetic dysregulation in rare pediatric brain tumors.

## 4 Toward therapies targeting epigenetic regulation

It has been recognized that pediatric brain tumors are often caused by epigenetic dysregulation. This growing understanding has opened new avenues for therapeutic intervention, and recent preclinical studies have demonstrated some promising strategies targeting epigenetic regulation (**Table 2**). These approaches aim to reverse or mitigate the epigenetic changes driving tumor growth, offering potential for more precise and effective treatments.

**Table 2:** Pre-clinical studies on treatments targeting epigenetic mechanisms.

Small molecule inhibitors	Tumor types	References
SMARCA4 inhibitor (BT869, SU-DIPGXIIIP)	DMG	(36)
NFIB inhibitor (TP064)	SHH-MB	(49)
HDAC inhibitor (Etinostat, Vorinostat)	MB (MYC-amplified)	(52, 53)
HDAC inhibitor (Trichostatin A)	SHH-MB^*1^ more effective in SHH-MB with *CREBBP* mutation	(55, 172)
HDAC inhibitor (Panobinostat)	IDH1-mutant glioma	(56)
HDAC inhibitor (quisinostat, romidepsin, etinostat, Panobinostat)	DMG	(58–60)
LSD1/HDAC inhibitor (Corin)	DMG	(59)
HDAC8 inhibitor (PCI34051)/EZH2 inhibitor (GSK126)	AT/RT	(29)
EZH2 inhibitor (UNC1999, GSK126, EPZ6438)	SHH-MB	(43)
EZH2 inhibitor (GSK343, EZP6438)	DMG	(146)
BMI inhibitor/ERK inhibitor	G4-MB	(37)
BMI inhibitor (PTC209)	DMG	(71)
Selective Serotonin Reuptake Inhibitor (SSRI)^*2^	ST-EPN-ZFTA	(145)
DNMT inhibitor (5-AzaC)/SMO inhibitor (sonidegib, vismodegib)	SHH-MB	(82)
DNMT inhibitor (5-AzaC)/HDAC inhibitor (vorinostat)	ETMR	(85)
DNMT inhibitor (decitabine)	AT/RT	(149)
LSD1 inhibitor (GSK-LSD1, ORY-1001)	MB (GFI1/MYC-driven)	(88)
GLI2 inhibitor (ATO)	ST-EPN-ZFTA	(92)
BET inhibitor (JQ1, OTX015)	MB (MYC-, MYCN-, GLI1/2-driven)	(173–176)
Thyroid hormone^*3^	SHH-MB	(177)

^*1^ more effective in SHH-MB with *CREBBP* mutation, ^*2^ inhibiting histone serotonylation, ^*3^ inhibiting EZH2 function.

One approach is the identification of molecules responsible for the epigenetic regulation of target genes followed by administering drugs that specifically act on these molecules. Recent successful examples are bromodomain-containing protein 4 (BRD4) inhibitor and thyroid hormone treatments. BRD4 is an epigenetic reader that recognizes histone acetylation motifs on ϵ-N-terminal lysine residues (178). At histone acetylated regions, BRD4 often promotes oncogene expression by interacting with various transcriptional factors and chromatin remodeling proteins, forming a bridge between super enhancer and promoter, and recruiting RNA polymerase II (179). In preclinical models of MB, JQ1 and OTX015, bromodomain and extraterminal (BET) inhibitors suppress oncogenic pathways in MBs driven by MYC (173, 174), GLI1 and GLI2 (175) and MYCN (176) via functional inhibition of BRD4. As the second example, thyroid hormone can inhibit EZH2 function by blocking the interaction between EZH2 and thyroid hormone receptor (TRα1). Thyroid hormone-mediated EZH2 inhibition reduces H3K27me3 histone marks at *NEUROD1* regulatory regions and enhances *NEUROD1* expression, eventually driving tumor cell differentiation into postmitotic cells and suppressing MB growth irreversibly (43, 177). These strategies hold promise as they target key epigenetic regulators directly, potentially enhancing therapeutic specificity and efficacy in cancer treatment.

The development of epigenetic drugs has consequently presented an attractive therapeutic approach in clinical setting. For example, HDAC inhibitors (e.g., Vorinostat, Panobinostat, Fimepinostat, and MTX110), EZH2 inhibitors (e.g., Tazemetostat), BMI inhibitors (PTC028, PTC596), and BET inhibitors (e.g., BMS-986158 and BMS-986378) are being utilized, and assessed their efficacy and safety in clinical trials (**Table 3**) (180–186). Although these therapies face issues such as poor blood-brain barrier (BBB) penetration, resistance due to tumor heterogeneity, and systemic toxicity, recent studies have been addressing these issues (185, 187, 188). However, challenges persist due to the lack of mechanistic clarity for individual cancer types, as well as off-target effects, which refer to the unintentional and broad impacts these drugs may have on non-target gene expression. These factors complicate efforts to mitigate side effects and address the lack of efficacy observed in some cases.

**Table 3:** Inhibitors used for clinical trials of epigenome-targeted therapy on pediatric brain tumors.

Small molecule inhibitors	Tumor types	NCT number	Phase	References
HDAC inhibitor				
Vorinostat Vorinostat + Temsirolimus*^1^	DMG DMG	NCT01189266 NCT02420613	I/II I	(180) (180, 181)
Panobinostat Panobinostat Panobinostat Panobinostat + Marizomib*^2^	DMG DMG AT/RT DMG	NCT02717455 NCT03632317 NCT04897880 NCT04341311	I I II I	(180, 182) (182) (183) (180)
Fimepinostat	DMG, HGG, MB	NCT03893487	I	(180, 182)
MTX110	DMG	NCT03566199	I/II	(182)
EZH2 inhibitor				
Tazemetostat Tazemetostat Tazemetostat	Solid tumors Solid tumors Solid tumors	NCT02875548 NCT02601950 NCT03155620	II II II	(184) (184) (182)
BMI inhibitor				
PTC028, PTC596	DMG, HGG	NCT03605550	I	(180)
BET inhibitor				
BMS-986158, BMS-986378	Solid tumors	NCT03936465	I	(180, 182)

*^1^mTOR inhibitor, *^2^proteasome inhibitor.

Please note that this table summarizes only clinical studies with epigenetic drugs that were introduced in this review.

Given that several genes are regulated by multiple chromatin regulatory factors, synthetic lethality approaches could be an alternative option to eliminate cancer by dysregulation of cancer epigenomes (189). Synthetic lethality occurs when the simultaneous impairment of two or more genes or pathways results in cell death, whereas the impairment of either one alone does not. This strategy leverages the unique vulnerabilities of cancer cells by targeting specific chromatin regulators in combination, aiming to induce cancer cell death while minimizing effects on normal cells. Furthermore, the synthetic lethality approach enables treatment of LOF mutations that were once considered undruggable (189). For example, loss of *SMARCB1* and inhibition of EZH2 cause synthetic lethality in malignant rhabdoid tumor (MRT) cells. EZH2 inhibition induces apoptosis, differentiation, and the expression of the tumor suppressor gene *p16^Ink4a^* specifically in *SMARCB1*-deficient MRT cells, due to epigenetic antagonism between SWI/SNF and PRC2 (190). In addition, HDAC inhibitors also cause synthetic lethality for *SMARCB1* loss. These inhibitors partially complement the histone acetylation function of the SWI/SNF complex in *SMARCB1*-deficient MRTs, enhancing the expression of differentiation markers and inhibiting the proliferation signaling of tumor cells (191, 192). Accordingly, EZH2 inhibitors and/or HDAC inhibitors are also applicable to *SMARCB1*-deficient AT/RT (29).

Synthetic lethality has also been explored in relation to the balance of histone acetylation. HDACs and HAT domain of CREBBP have opposing functions; therefore, HDAC inhibitors complement HAT activity, which makes them more effective in SHH-MB with *CREBBP* mutation than *CREBBP* wild-type (172). Alternatively, interaction between *CREBBP* loss and p300 inhibition also induces synthetic lethality through distinct mechanisms. Both CREBBP and p300 have HAT activity; hence, p300 inhibitors can induce synthetic lethality for *CREBBP*-deficient tumor cells by inhibiting compensatory p300 activation in lung and hematopoietic cancer cells through the regulation of MYC promoter activity (193). A similar effect of p300 inhibitors could also be expected in *CREBBP*-deficient MBs. Thus, synthetic lethality-based functional screening would be one of the powerful approaches for identification of druggable targets.

A recent novel sophisticated approach for seeking a potential cancer therapy is the use of CRISPRi to precisely target epigenetic loci. This method involves expressing histone regulatory proteins, such as p300 or Krüppel-Associated Box (KRAB), fused with dead CRISPR-associated nuclease 9 (dCas9) and recruiting these complexes to the regulatory regions of target genes, specifically promoter regions, via single-guide RNA (sgRNA), leading to either activation by p300 or repression by KRAB of gene expression (49, 194, 195). Unlike the synthetic lethality approach, this method specifically targets the expression of genes critical to cancer proliferation or cell death, potentially reducing side effects. However, there are still several challenges associated with CRISPR/dCas9 such as off-target effects, delivery efficiency, lack of persistence due to instability, and cancer resistance. Recent studies have been addressing these issues through improved sgRNA design with high-fidelity dCas9 variants, efficient and stable delivery systems (e.g., viral vectors, lipid nanoparticles), and combination therapy with immunotherapy and molecular targeted drugs (196). Further research to overcome these challenges will be essential for advancing CRISPR/dCas9-based therapies toward clinical applications.

## 5 Conclusion

Over the past few decades, epigenetic dysregulation has been increasingly recognized as a critical factor in the tumorigenesis of pediatric brain tumors. Chromatin regulatory dysfunction, with proper timing and regional specificity, plays a pivotal role in diverting normal neuronal differentiation signaling toward oncogenesis. Insights into epigenetically driven tumorigenesis could pave the way for novel treatments that shift oncogenic pathways back toward normal differentiation signaling. The chromatin modifications underlying tumor development consist of various mechanisms, including mutation or dysregulation of chromatin remodelers, histone modifiers, histones themselves, and DNA methyltransferases. Genomic rearrangement events disrupt epigenetic regulation by generating new fusion genes or placing existing genes in new regulatory contexts. These mechanisms are commonly seen in multiple brain tumor types, and elucidating functional similarities and differences across tumor types may provide clues to finding new common therapeutic approaches. Indeed, certain targeted therapies could even be applied to multiple tumor types as seen in HDAC inhibitors for SHH-MB, MYC-amplified G3-MB, and DMG (52–55, 58–60, 137, 172). Furthermore, the impact of tumor microenvironment on epigenetics has been gaining attention as an emerging field. As seen in the histone modification by neurotransmitters (e.g., histone serotonylation), other elements of tumor microenvironment may also regulate chromatin states and could serve as druggable targets. When considering potential treatments targeting epigenomes, incorporating the latest knowledge (e.g., synthetic lethality) and cutting-edge technologies (e.g., CRISPR/dCas9) into therapeutic strategies may also be crucial for maximizing the efficacy while minimizing the side effects. In conclusion, further multifaceted studies will be required to elucidate the epigenetic mechanisms and advance innovative therapies for pediatric brain tumors.
